# Effect of Danhong injection on heart failure in rats evaluated by metabolomics

**DOI:** 10.3389/fmed.2023.1259182

**Published:** 2023-10-04

**Authors:** Lin Li, Senjie Zhong, Jiahao Ye, Siyuan Hu, Zhixi Hu

**Affiliations:** ^1^The Domestic First-class Discipline Construction Project of Chinese Medicine, Hunan University of Chinese Medicine, Changsha, Hunan, China; ^2^Provincial Key Laboratory of TCM Diagnostics, Hunan University of Chinese Medicine, Changsha, Hunan, China; ^3^Hunan Engineering Technology Research Center for Medicinal and Functional Food, Changsha, Hunan, China; ^4^The First Affiliated Hospital of Guangzhou University of Chinese Medicine, Guangzhou, Guangdong, China; ^5^Post-Graduate School, Hunan University of Chinese Medicine, Changsha, Hunan, China

**Keywords:** heart failure, Danhong injection, metabolomics, transverse aortic constriction, Chinese medicine (CM)

## Abstract

**Background:**

Heart failure (HF) is characterized by reduced ventricular filling or ejection function due to organic or non-organic cardiovascular diseases. Danhong injection (DHI) is a medicinal material used clinically to treat HF for many years in China. Although prior research has shown that Danhong injection can improve cardiac function and structure, the biological mechanism has yet to be determined.

**Methods:**

Serum metabolic analysis was conducted via ultra-high-performance liquid chromatography-quadrupole time-of-flight/mass spectrometry (UHPLC-QE/MS) to explore underlying protective mechanisms of DHI in the transverse aortic constriction (TAC)-induced heart failure. Multivariate statistical techniques were used in the research, such as unsupervised principal component analysis (PCA) and orthogonal projection to latent structures discriminant analysis (OPLS-DA). MetaboAnalyst and Kyoto Encyclopedia of Genes and Genomes (KEGG) were employed to pinpoint pertinent metabolic pathways.

**Results:**

After DHI treatment, cardiac morphology and function as well as the metabolism in model rats were improved. We identified 17 differential metabolites and six metabolic pathways. Two biomarkers, PC(18:3(6Z,9Z,12Z)/24:0) and L-Phenylalanine, were identified for the first time as strong indicators for the significant effect of DHI.

**Conclusion:**

This study revealed that DHI could regulate potential biomarkers and correlated metabolic pathway, which highlighted therapeutic potential of DHI in managing HF.

## 1. Introduction

Heart failure (HF) is characterized by reduced ventricular filling or ejection function due to organic or non-organic cardiovascular diseases (1). It is a severe manifestation or end stage of a variety of cardiovascular diseases, with significant morbidity and mortality (2). The fundamental cause of HF is multifaceted and incompletely understood, which limits the development and availability of specific therapies. Therefore, further study on the pathogenesis and therapeutic targets for HF is urgently needed.

Traditional Chinese Medicine (TCM) has remarkable advantages in managing HF for its efficacy but with fewer side effects (3). It has distinct advantages in alleviating HF clinical symptoms, improving quality of life, and preventing disease progression (4). Danhong injection (DHI), a Chinese patent compound injection, is widely used in the prevention and treatment of various cardiovascular diseases, including reperfusion injury, atherosclerosis, and acute coronary syndrome, among others, since its introduction to the market in 2002 (5–7). Systematic review validates the clinical efficacy and safety of DHI in the treatment of CHF (8). It consists of two components, the roots of *Salvia miltiorrhiza Bge*(Danshen) and the flowers of *Carthamus tinctorius L*(Honghua) (9). The effective ingredients of DHI mainly include tanshinone, salvianolic acid, and danshenxin quinone. The rationale behind investigating DHI lies in its unique properties and mechanisms of action that make it relevant to cardiovascular and cerebrovascular disease management (10). Previous studies have shown that DHI possesses anti-inflammatory, antioxidant, and antithrombotic properties (11, 12), which are crucial in mitigating the pathological processes underlying heart failure. It could alleviate cardiac fibrosis by preventing the hypermethylation of Rasal1 and Rassf1 in TAC mice, and enhance angiogenesis after myocardial infarction by activating the MiR-126/ERK/VEGF pathway (13), and shown protective effects for cardiomyocytes (14). Network pharmacology analysis and experimental validation also showed that DHI attenuates doxorubicin-induced cardiotoxicity in rats via suppression of apoptosis (15). Furthermore, its ability to improve cardiac function, reduce myocardial injury, and enhance vascular endothelial function has been reported in both animal and clinical studies (8, 16).

Despite these promising findings, the exact molecular mechanisms and metabolic changes associated with Danhong injection’s effects on heart failure remain largely unexplored. Metabolomics can help identify specific metabolites or metabolic pathways that are altered in heart failure and may be influenced by Danhong injection. These biomarkers can serve as valuable indicators of treatment efficacy and disease progression. Danielle used metabolomics on blood from the artery, coronary sinus, and femoral vein in 110 patients with or without heart failure to quantify the uptake and release of 277 metabolites (17). Zhou et al. use plasma metabolomic and lipidomic profiles in different HF stages to identify potential biomarkers (18). Therefore, a comprehensive assessment using advanced analytical techniques is necessary to elucidate the metabolic alterations induced by Danhong injection in heart failure and provide insights into its potential therapeutic benefits.

The pathological process of HF might be ascribed to various metabolic disturbances, particularly the aberrant levels of cardiac energy factors including amino acids, glucose, fatty acids, and ketone bodies (19). Long-term metabolic dysfunction could substantially disrupt cardiac energy metabolism and deteriorate cardiac function. Thus, maintaining appropriate metabolic homeostasis in the heart appears to be a viable approach to treating HF. Animal model is the basic material of HF research, and transverse aortic constriction (TAC) is an ideal method for replicating HF models (20). The increased ventricular afterload caused by arteriosclerosis or hypertension can be properly recapitulated by TAC, which will gradually progress into HF. Studying the effects of TCM treatments on HF utilizing a stable HF animal model system will help to further clarify the pathological mechanism and therapeutic targets of heart failure, which might lead to more breakthroughs for heart failure treatment. Although prior research has shown that Danhong injection can improve cardiac function and structure in the TAC model (21), the underlying biological mechanism has yet to be determined.

In the current study, the non-targeted metabolomics technology of UHPLC-QE-MS will be utilized to detect any change in serum metabolites and assess associated metabolic pathways in DHI-treated rats with TAC-induced HF for the first time, our results will provide a better understanding of the metabolic therapeutic mechanisms of Danhong injection in the TAC model, as well as to offer an experimental foundation for the management of HF and the rational use of TCM injection.

## 2. Materials and methods

### 2.1. Heart failure model and treatment

Sprague–Dawley (SD) rats (male, 200–220 g, *n* = 32), provided from the Hunan SJA Laboratory Animal Co., Ltd. (License number: SCXK(Xiang)2019–0004), were kept in a controlled environment that is free of specific pathogens can be created by implementing a lighting schedule consisting of 12 h of light and 12 h of darkness, while also regulating the surrounding temperature to be kept at 24 ± 2°C.

All groups were given a normal diet (hydration≤10%, crude protein≥18%, crude fat≥4%, crude fiber ≤5%, calcium 1.0%–1.8%). These rats were housed in cages (3 rats per cage), and 60 g of feed were fed to each cage every day, and free drinking water. The animal-related procedures were monitored and authorized by the Institutional Animal Care and Use Committee of the Hunan University of Chinese Medicine (no: LL2021011302).

Random allocation was performed on SD rats after one week of adaptation, with 8 assigned to the sham group and 24 to the TAC group. TAC was used to establish the HF model. These methods were based on previous literature (22, 23). Rats in the TAC group were anesthetized by urethane (1.0 g·kg^−1^) via i.v. administration, and tracheal cannula (tidal volume = 6–8 mL/kg, frequency = 80 times/min) was utilized to maintain breathing. The thoracic cavity was opened, the 2nd-3rd intercostal space was bluntly dissected, the aortic arch was located, and the 4–0 coarctation line was passed under the aortic arch and pulled out the space situated amidst the left common carotid artery and the right innominate artery. The self-made constriction pin was put parallel to the aortic arch after the aortic arch was raised. The pin was rapidly withdrawn after full occlusion, and the aortic arch was constricted to less than 70% of its original outside diameter. After that, the chest cavity closure and skin suture were performed followed by turning off ventilator. To avoid infection, penicillin sodium was injected 3 days after the operation. Sham surgery without constriction of aortic arch was performed as a sham operation. The rats were kept for two months (60 days) after the operation.

After 60 d of feeding, the rats of the TAC were randomly stratified to three groups (8 per group): TAC group, Danhong injection (DHI) group and Trimetazidine treatment (TMZ) group. DHI group rats were treated daily with intraperitoneal injections of Danhong (6.0 mL·kg^−1^) and gavage with normal saline (4.0 mL·kg^−1^). TMZ group rats were treated daily with intraperitoneal injections of sterile injection water (6.0 mL·kg^−1^) and gavage with Trimetazidine (dissolve in normal saline, 10 mg·kg^−1^). Rats (both sham and TAC groups) were administrated daily with intraperitoneal injections with sterile water (6.0 mL·kg^−1^) and gavage with normal saline (4.0 mL·kg^−1^). The chemicals were administered for 15 days. Shandong Danhong Pharmaceutical Co., Ltd. (Shandong, China, Catalog no. 20112012) generously provided DHI. Trimetazidine hydrochloride was purchased from Rayon Pharmaceutical Co., Ltd. (Shangdong, China, Catalog No. 20121706).

### 2.2. Echocardiography evaluation

To prepare for the echocardiography assessment, urethane (1.0 g·kg-1) was utilized to anesthetize the rats followed by evaluation by SonoScape-S2N ultrasound system (Shenzhen Kaili technology co., Ltd.). Two-dimensional images were used for measuring the parameters and M-mode interrogation was conducted. Using the Teichholtz formula, we calculated the left ventricular ejection fraction (LVEF) and left ventricular fraction shortening (LVFS).

### 2.3. Sample collection and preparation

Urethane anesthesia (1.0 g/kg, i.p.) was administered to the rats. Blood and heart tissue samples were obtained under anesthetization, and cervical dislocation was performed to sacrifice the rats when the experiments concluded. Blood samples were obtained via abdominal aorta followed by removal of the clot by centrifugation (3,000 rpm for fifteen minutes at 4°C). Serum levels of NT-proBNP were assessed by ELISA kits (Wuhan, China). Subsequently, serum collection was achieved via rapidly frozen using liquid nitrogen and maintained at −80°C for metabolomic evaluation. Following fixation using 4% PFA solution, we embedded the heart tissues in paraffin blocks, from which we then sectioned the block at 5-μm. Hematoxylin and eosin staining was conducted on tissue slides (Microm HM325, Thermo).

### 2.4. UHPLC-QE-MS conditions

For the LC–MS/MS assessment, a UHPLC system from ThermoFisher (Vanquish, Thermo Fisher Scientific, United States) was used in conjunction with a Q Exactive HFX (Thermo Fisher Scientific, United States) mass spectrometer from the same manufacturer and a UPLC BEH Amide column measuring 2.1 mm × 100 mm with a 1.7 μm particle size. The mobile phase consisted of two components: a solution of 25 mmol/L ammonium acetate and 25 ammonia hydroxide in water with a pH of 9.75 (component A), and acetonitrile (component B). Gradient elution (0–0.5 min, 95% B, 0.5–7 min, 95-65%B, 7–8 min, 65%B–40%B, 8-9 min, 40%B; 9–9.1 min, 40-95%B; 9.1–12 min, 95%B), the auto-sampler temperature was maintained at 4°C and the injection volume was set to 2 μL.

The QE HFX mass spectrometer used in this study was equipped to capture MS/MS spectra while operating in information-dependent acquisition (IDA) mode, which was controlled by the acquisition software (ThermoFisher). During IDA mode, the acquisition software continuously monitored the fully scanned MS spectrum. The ESI source was configured with a sheath gas flow rate of 30 Arb, an Aux gas flow rate of 25 Arb, and a capillary temperature of 350°C. In addition, the MS resolution was set at 120,000, the MS/MS resolution at 7,500, and the collision energy at 10/30/60 in NCE mode. Finally, the spray voltage was either set to 3.6 kV (positive) or − 3.2 kV (negative).

### 2.5. Sample preparation methodology

100 μL of sample was transferred to an EP tube. After the addition of 400 μL ofextract solution (acetonitrile: methanol = 1: 1, containing isotopically-labelled internal standard mixture), the samples were vortexed for 30 s, sonicated for 10 min in ice-water bath, and incubated for 1 h at −40°C to precipitate proteins. Then the sample was centrifuged at 12000 rpm(RCF = 13,800(×g),R = 8.6 cm) for 15 min at 4°C. The resulting supernatant was transferred to a fresh glass vial for analysis.

We pooled the all samples extract together for quality control (QC) preparation, which was to evaluate the deviations generated from the results of pooled mixtures followed by comparison to the instrument-derived errors. As illustrated in Supplementary Figure S1, the instrument utilized in this study is of high stability and reproducibility, as evidenced by the clear cluster in QC samples.

### 2.6. UHPLC-QE-MS result processing and statistical analysis

Result processing was done according to previous publication (24). To process the raw data, the ProteoWizard software was used to transform it to mzXML format. The resulting data was then subjected to peak detection, extraction, alignment, and integration using an R-based program developed on XCMS. BiotreeDB was utilized to carry out metabolite annotation (cutoff value = 0.3). Results were obtained in positive and negative ion modes, which were then combined for analysis. The LC–MS detection was supported by Biotree company in Shanghai, China.

To perform multivariate analysis of the Metabolomics dataset, SIMCA16.0.2 software package (Umea, Sweden) was utilized by importing peak number, sample name, and normalized peak area. PCA and OPLS-DA are frequently adopted for multivariate analysis. Then, seven-fold cross-validation was conducted to obtain the value of R2 and Q2. A VIP value > 1.5 and a fold-change ratio of ≥1.2 or ≤ 0.83 (OPLS-DA model) with *p* < 0.05 in the student’s *t*-test were set as the thresholds for screening potential metabolites with significant differences. The makers were entered into MetaboAnalyst to determine the impacted metabolism related pathways.^1^ The Data analysis method is consistent with the previous publication (16).

### 2.7. Statistical analysis

To perform statistical analysis, the software IBM SPSS Statistics 22.0 (Chicago, United States) was utilized. One-way ANOVA was utilized to assess the variations between groups. *p* < 0.05 was adopted as significance threshold for all tests.

## 3. Results

### 3.1. Quality control of Danhong injection

**Figure 1:**
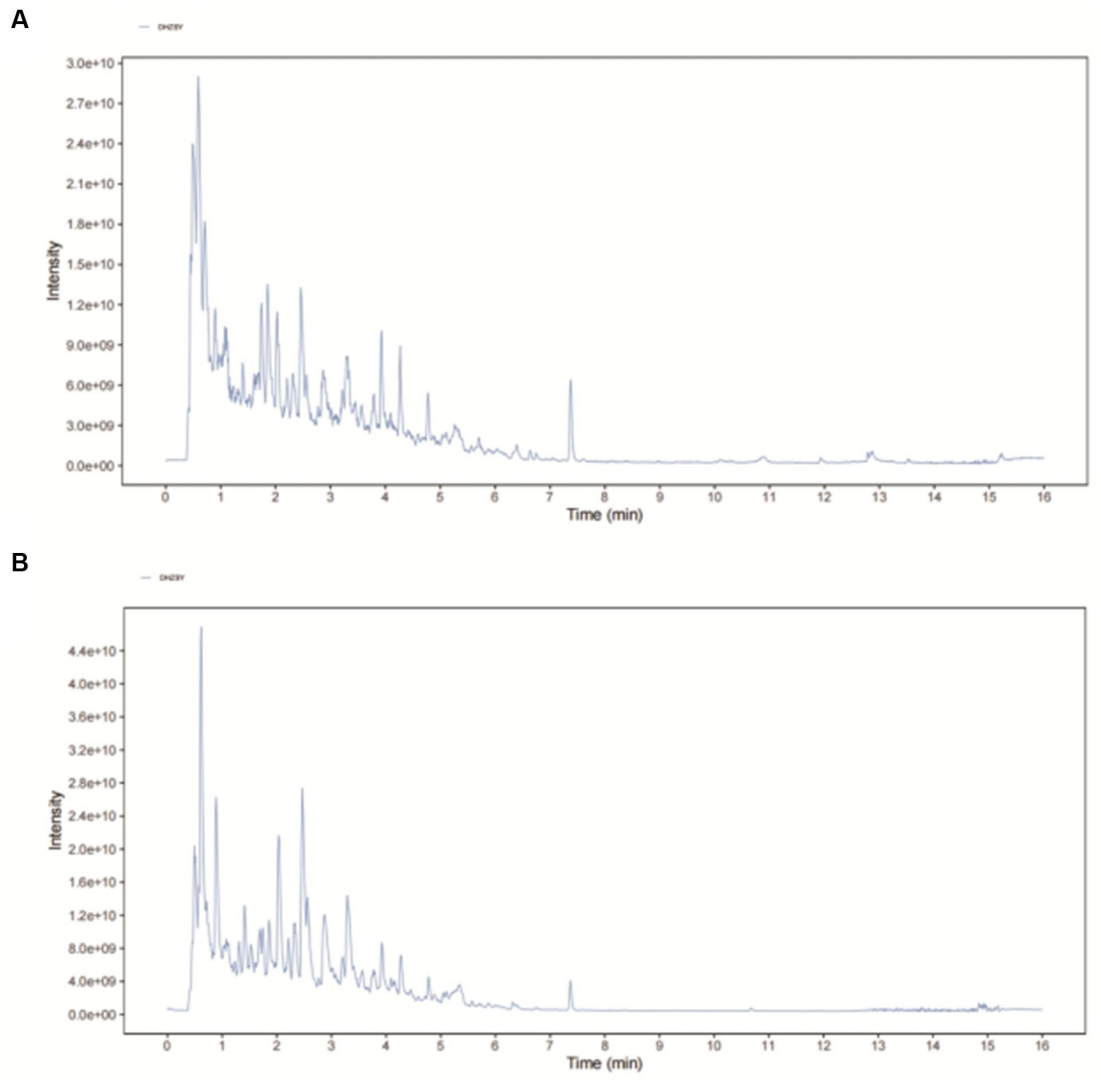
The representative UHPLC-QE-MS chromatogram of DHI. **(A)** Positive mode and **(B)** Negative mode.

Following the corresponding quality control standard, an established method was employed to conduct DHI quality control through UHPLC-QE-MS analysis (25). A representative UHPLC-QE-MS chromatogram from one of the samples of DHI is shown in Figure 1, and the relative information to primary compounds is exhibited in Supplementary Table S1. A superimposed plot of the top 10 effective components and standard substances is in Supplementary Figure S2.

### 3.2. Danhong injection improved cardiac function in rats with TAC-induced HF

**Figure 2:**
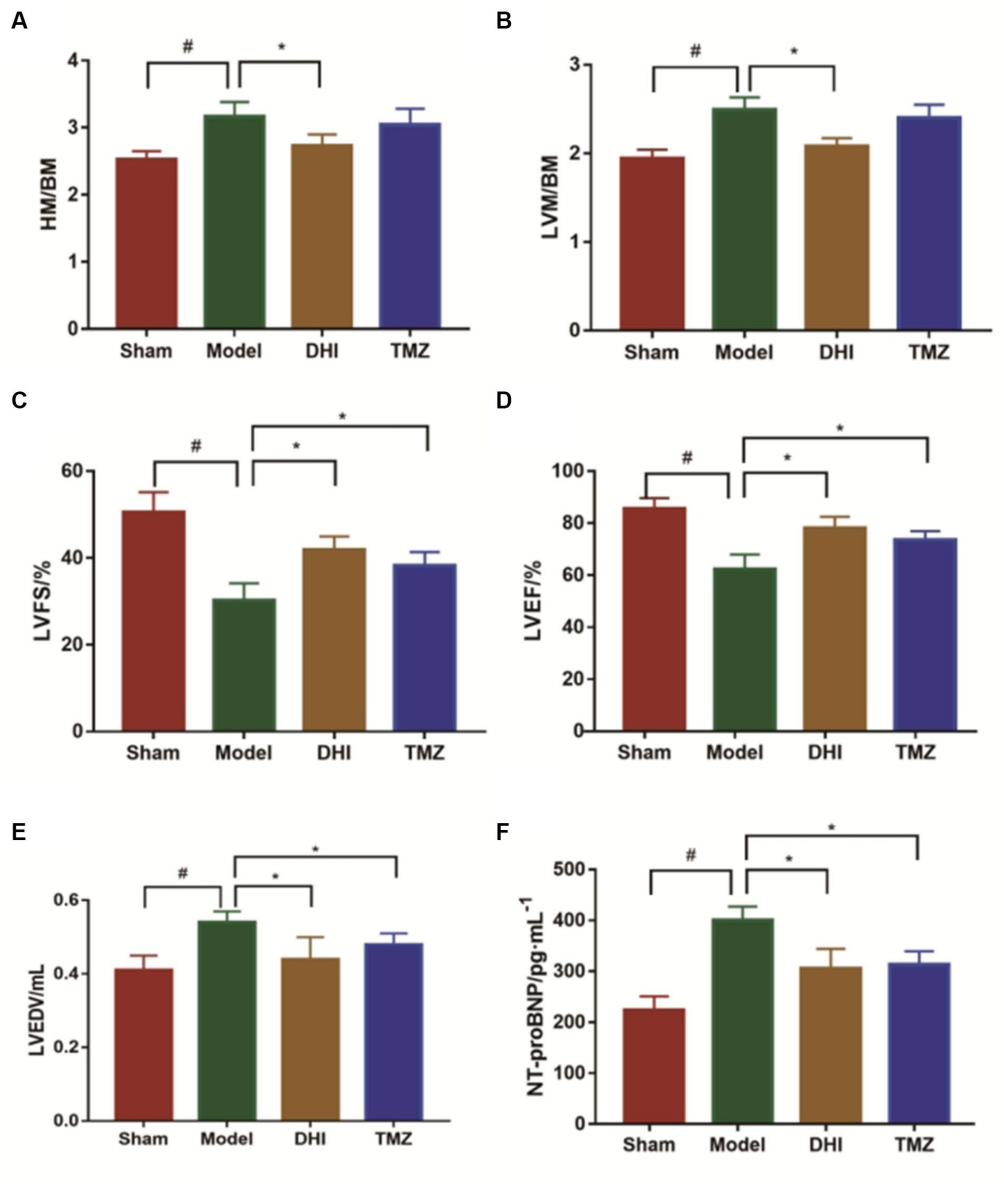
Evaluation of the cardiac function. **(A)** Heart mass to body mass ratio (HM/BM); **(B)** Left ventricular mass to body mass ratio (LVM/BM); **(C)** Left ventricular fractional shortening (LVFS); **(D)** Left ventricular ejection fraction (LVEF); **(E)** left ventricular end-diastolic volume (LVEDV); and **(F)** NT-proBNP was measured by ELISA. #compare with sham group, *to model group, *p* < 0.05. 8 in each group.

**Figure 3:**
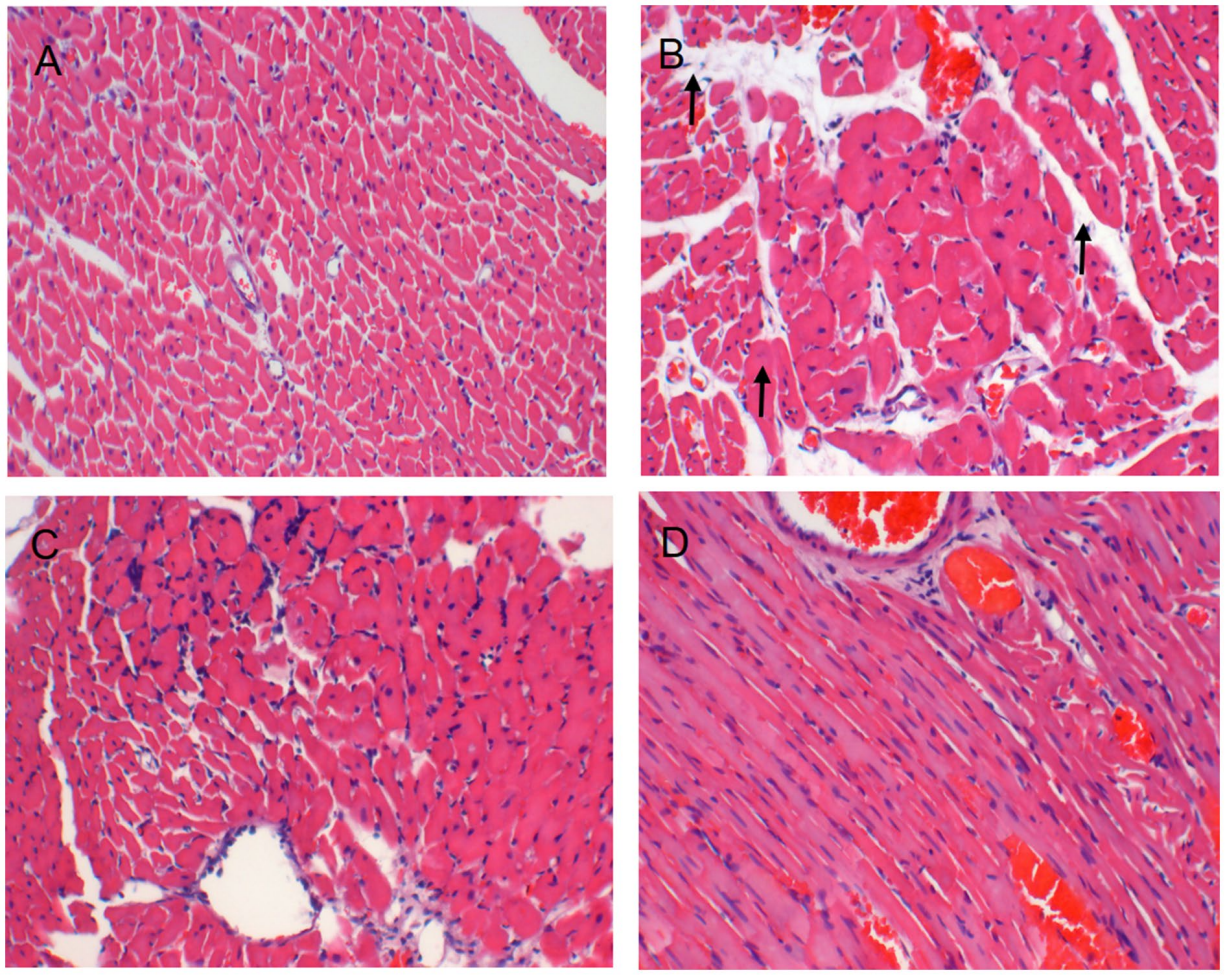
HE staining images of histological changes in the heart (**A, B, C, D**: × 200), **(A)** Sham group; **(B)** Model group; **(C)** DHI group; and **(D)** TMZ group.

Serum levels of NT-proBNP were upregulated in the TAC rats than in the controls (*p* < 0.05), suggesting that HF model was successfully established (Figure 2F). Results from HE staining clearly showed increased infiltration of immune cells and noticeable cardiomyocyte hypertrophy, resulting in disarrangement of myocardial cells following TAC, when comparing with controls (Figure 3). More importantly, cardiac function was notably aggravated in model group, as supported by reduced LVEF/LVFS and increased LVEDV, when comparing to sham group. The histological and cardiac function results were consistent with NT-proBNP levels (Figure 2). Nonetheless, LVEF and LVFS were significantly increased whereas LVEDV and NT-proBNP were significantly decreased in rats treated with DHI or TMZ. Results of HE staining showed more organized arrangement of cardiomyocytes with clear cross-striations, and collagen fiber deposition was alleviated in the DHI group and TMZ group. Lastly, administration of DHI has shown to alleviate cardiac hypertrophy, as evidenced by decreased heart weight/ left ventricle weight to body weight ratios (Figures 2A,B).

### 3.3. DHI improves metabolism disorder

#### 3.3.1. Metabolite profiling analysis

**Figure 4:**
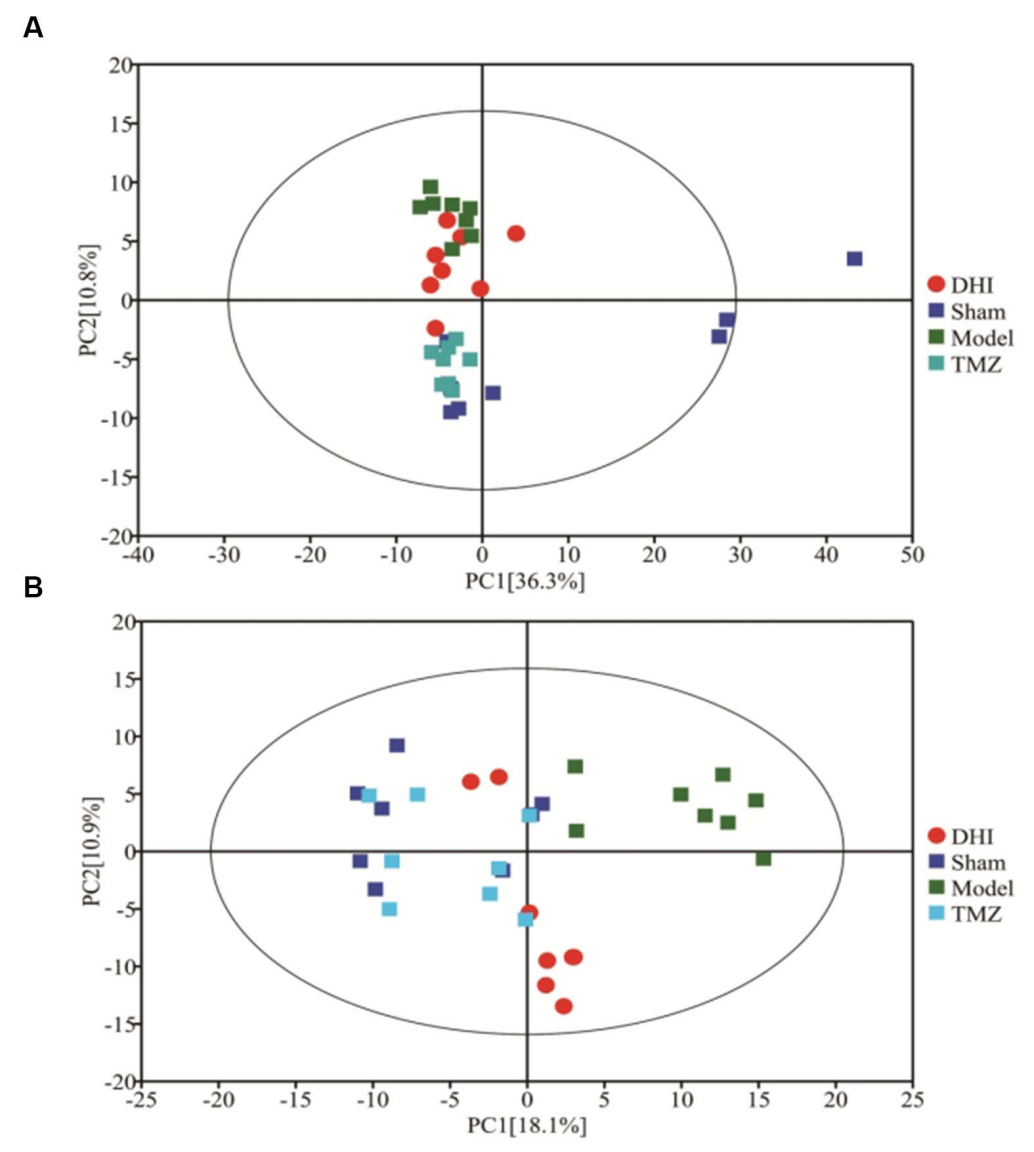
PCA score of four groups. **(A)** PCA score plots for positive-ion mode (R^2^X = 0.576); **(B)** PCA score plots for negative-ion mode (R^2^X = 0.413).

The profile of altered metabolites was detected using principal component analysis (PCA). Figure 4A shows that the scatter data points were clearly segregated between the sham and model groups. After treatment, the data points from DHI group and TMZ group were grouped more closely with data points from the Sham group, indicating that the metabolic profile was normalized after the intervention of the two drugs.

#### 3.3.2. DHI improves the metabolites disorder

**Figure 5:**
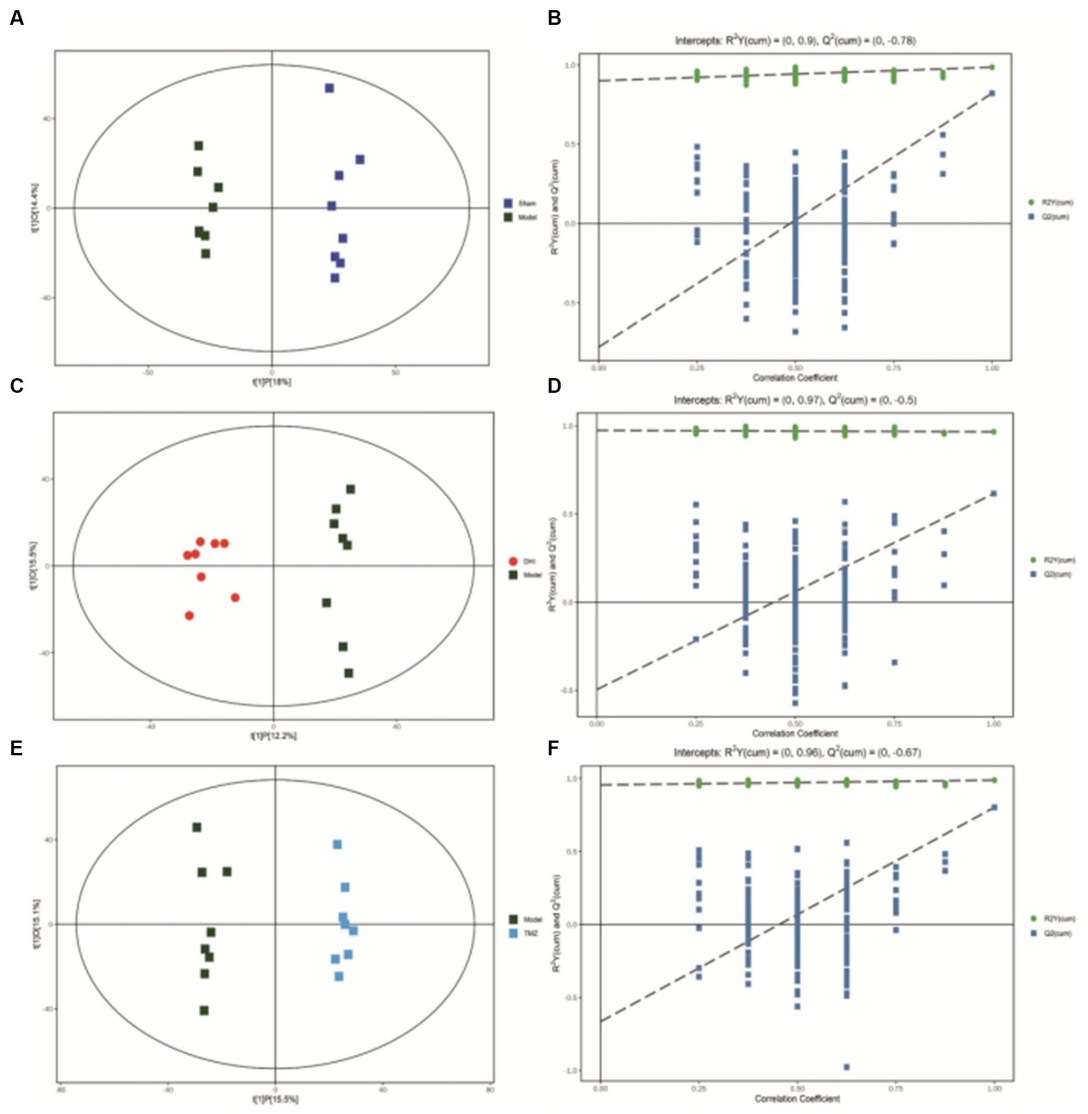
**(A)** OPLS-DA scores comparison plots in the positive-ion mode for Sham and Model groups (R2X = 0.324, R2Y = 0.986, Q2 = 0.822). **(B)** Permutation test (*n* = 200) for comparing the OPLS-DA model of Sham and Model groups in positive-ion mode. **(C)** OPLS-DA score plots in the positive-ion mode for Model and DHI groups (R2X = 0.276, R2Y = 0.966, Q2 = 0.616). **(D)** Permutation test (*n* = 200) for comparing OPLS-DA model of Model and DHI groups in positive-ion mode. **(E)** OPLS-DA score plots in the positive-ion mode for Model and TMZ groups (R2X = 0.306, R2Y = 0.989, Q2 = 0.803). **(F)** Permutation test (*n* = 200) for comparing OPLS-DA model of Model and TMZ groups in positive-ion mode.

**Figure 6:**
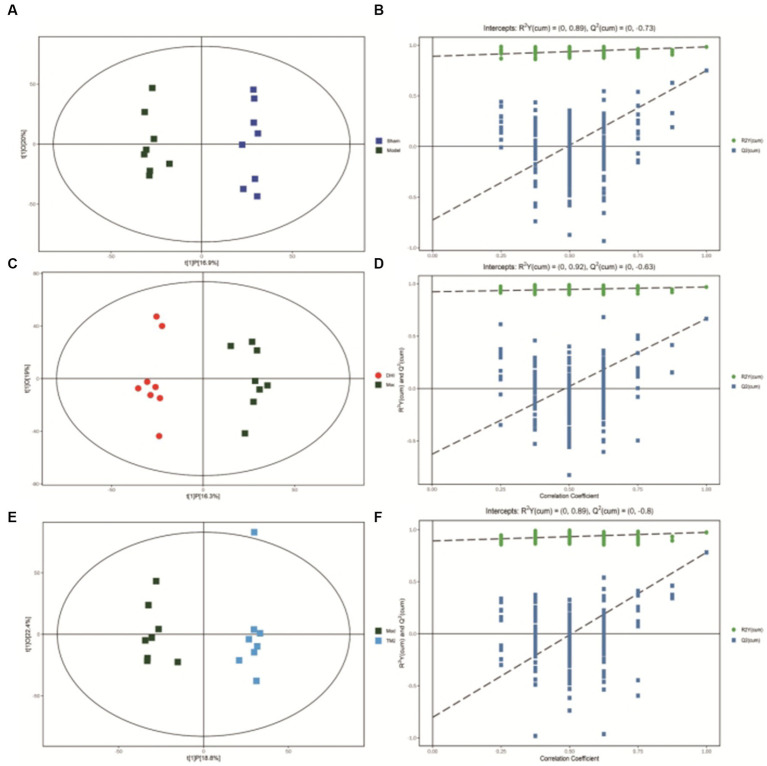
**(A)** OPLS-DA scores comparison plots in the negative-ion mode for Sham and Model groups (R2X = 0.369, R2Y = 0.983, Q2 = 0.75). **(B)** Permutation test (*n* = 200) for comparing the OPLS-DA model of Sham and Model groups in negative-ion mode. **(C)** OPLS-DA score plots in the negative-ion mode for Model and DHI groups (R2X = 0.353, R2Y = 0.968, Q2 = 0.666). **(D)** Permutation test (*n* = 200) for comparing OPLS-DA model of Model and DHI groups in negative-ion mode. **(E)** OPLS-DA score plots in the negative mode for Model and TMZ groups (R2X = 0.412, R2Y = 0.973, Q2 = 0.781). **(F)** Permutation test (*n* = 200) for comparing OPLS-DA model of Model and TMZ groups in negative-ion mode.

After scaling and log-transforming the data to reduce the impact of both noise and high variance, the Orthogonal Partial Least Squares Discriminant Analysis (OPLS-DA) was utilized to effectively distinguish among the four groups (Figures 5, 6). A VIP value > 1.0 and a fold-change ratio of ≥1.2 or ≤ 0.83 (OPLS-DA model) with *p* < 0.05 were adopted for the thresholds to screen potential metabolites with significant differences. In comparison to sham group, the expression of 160 metabolites were changed in model group. Among these metabolites, the level of 112 metabolites was increased and that of 48 metabolites was decreased. Since such a large number of differential metabolites was preliminarily screened, some metabolites are not included in the database and cannot be classified, or their functions, molecular structures, and associated pathways are not clear. To improve the accuracy and specificity of differential metabolites, the standard of increased calorie value was VIP > 1.5. In addition, we screened these metabolites using the KEGG and HMDB databases to identify targets that were highly relevant to TAC model and sought out to further assess the therapeutic potential of DHI.

**Figure 7:**
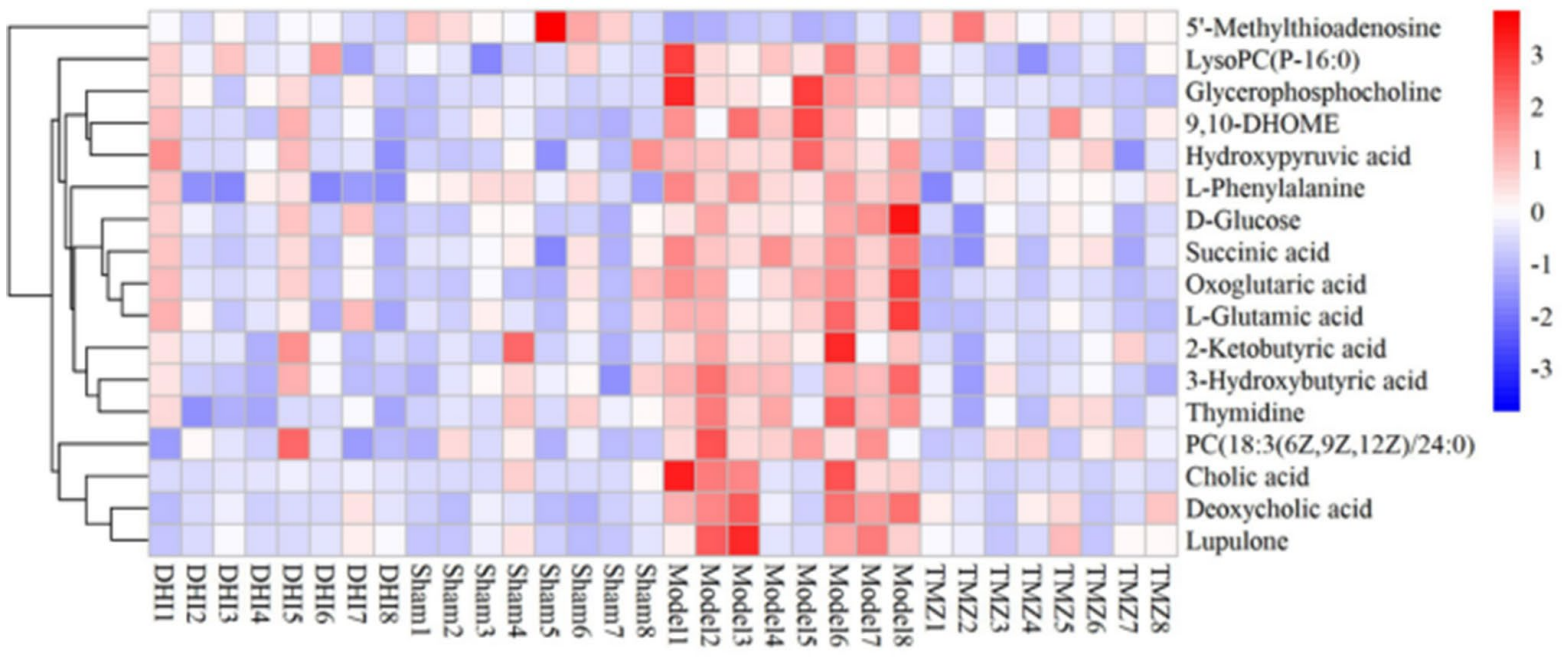
Heat map of the differential metabolites.

In comparison to sham, the levels of 17 metabolites in the DHI group were altered, including Cholic acid, 5′-Methylthioadenosine, Glycerophosphocholine, LysoPC(P-16:0), PC(18:3(6Z,9Z,12Z)/24:0), 3-Hydroxybutyric acid, D-Glucose, Deoxycholic acid, L-Phenylalanine, Thymidine, Succinic acid, 2-Ketobutyric acid, Oxoglutaric acid, Hydroxypyruvic acid, L-Glutamic acid, 9,10-DHOME, Lupulone. In comparison to model, the levels of 12 metabolites in the TMZ group were differentially reversed, and the other 5 metabolites did not meet the screening criteria (Tables 1, 2). A heatmap showing differential metabolites is shown in Figure 7.

**Table 1:** Basic information on differential metabolites.

No	Metabolites	M/z	Retention time/s	KEGG id	Ion mode	MS2
1	Cholic acid	431.27	239.52	C00695	POS	58.065627;59.073558;60.080987;104.106877;105.110991
2	5′-Methylthioadenosine	298.09	88.68	C00170	POS	53.83006;61.011616;75.026057;76.7149;87.044845;97.029534;122.002215;136.062807;137.063534;145.031944;163.042747;298.094554
3	Glycerophosphocholine	258.11	402.70	C00670	POS	54.433925;55.881005;58.066643;60.081726;64.524822;71.073553;72.450883;83.233023;86.09673;104.106728;124.999405;184.075867;258.112402
4	LysoPC(P-16:0)	480.34	208.00	C04230	POS	53.368224;55.055029;56.050047;57.03434;58.065689;60.081192;71.074089;86.097438;98.984933;104.107366;124.999972;181.025242;184.071057;240.098925;480.344629;481.340496
5	PC(18:3(6Z,9Z,12Z)/24:0)	868.67	156.06	C00157	POS	71.073312;77.005769;86.097602;86.311723;87.044831;89.059643;96.512266;98.984335;104.107822;125.000181;133.085644;183.338115;184.076081;185.077418;186.076797;217.150736;302.260044;810.617008;868.683565
6	3-Hydroxybutyric acid	103.03	221.04	C01089	NEG	57.033837;103.03938
7	D-Glucose	179.05	234.50	C00221	NEG	59.012568;71.013105;87.007426;89.023322;90.027745;106.935503;107.584332;107.93305;112.051196;122.893788;135.045412;135.96333;178.813032;178.924298;179.070834;179.954003
8	Deoxycholic acid	391.28	133.07	C04483	NEG	51.284937;51.88721;59.013591;96.320883;105.065464;214.229063;235.07569;302.329106;306.480302;343.273987;345.276693;391.285989
9	L-Phenylalanine	164.07	282.14	C00079	NEG	50.824324;57.586565;66.008792;72.007632;74.023896;75.007334;91.0537;93.03368;103.055228;120.045096;121.027959;121.998056;147.045652;148.046007;164.070449;164.835727;165.072592
10	Thymidine	241.08	90.42	C00214	NEG	51.240013;59.012477;60.273656;61.987449;79.955841;96.959419;125.033479;151.050753;161.035755;197.15608;198.074746;241.086074;241.227389;242.174973
11	Succinic acid	117.01	424.55	C00042	NEG	73.028673;88.018207;91.029831;99.008676;99.924654;100.924951;116.259135;116.926935;117.927016
12	2-Ketobutyric acid	101.02	93.82	C00109	NEG	55.017903;59.013394;71.013108;73.028647;83.012681;100.944486;101.060275
13	Oxoglutaric acid	145.01	389.58	C00026	NEG	77.893314;99.924709;100.925533;101.060203;101.92326;118.028837;144.913736;145.044936;146.023862
14	Hydroxypyruvic acid	103.00	430.72	C00168	NEG	53.78872;55.639518;57.034105;59.013347;60.016241;63.125294;68.800667;73.028146;102.92267;103.039165
15	L-Glutamic acid	146.04	418.18	C00025	NEG	53.950882;57.034101;57.975145;61.987458;62.13548;74.790194;85.028995;101.023312;101.923252;102.056017;103.039355;108.738947;128.034963;129.017681;146.044106;147.018108
16	9,10-DHOME	313.23	63.40	C14828	NEG	50.384481;55.18688;58.004742;59.012612;77.148753;78.30384;78.917474;79.955835;80.916169;91.020541;99.081107;129.08996;169.846841;183.137381;277.211051;295.231449;313.237882;313.691819;314.238723
17	Lupulone	413.26	171.19	C10706	NEG	59.012618;81.935505;82.500611;120.342217;167.443383;203.880229;213.724962;233.612036;255.232332;302.351686;395.254641;413.273896;414.273744

MS, mass spectrometry; m/z, mass-to-charge ratio; NEG, negative ion; POS, positive ion; RT, retention time.

**Table 2:** Regulation effects of Danhong injection on serum metabolites of TAC rats.

No	Metabolites	Model vs. Sham			DHI vs. Model			TMZ vs. Model		
		VIP	FC	Trend	VIP	FC	Trend	VIP	FC	Trend
1	Cholic acid	1.60	5.46	↑	1.83	0.15	↓	2.00	0.07	↓
2	5′-Methylthioadenosine	2.02	0.30	↓	1.94	1.76	↑	2.15	2.68	↑
3	Glycerophosphocholine	2.00	2.51	↑	2.09	0.54	↓	2.20	0.38	↓
4	LysoPC(P-16:0)	1.67	1.36	↑	1.73	0.81	↓	2.03	0.69	↓
5	PC(18:3(6Z,9Z,12Z)/24:0)	1.77	2.41	↑	1.63	0.48	↓	–	–	–
6	3-Hydroxybutyric acid	1.59	1.50	↑	1.83	0.63	↓	1.79	0.60	↓
7	D-Glucose	1.92	1.64	↑	1.60	0.71	↓	1.77	0.60	↓
8	Deoxycholic acid	1.94	4.70	↑	1.78	0.31	↓	–	–	–
9	L-Phenylalanine	1.65	1.50	↑	1.90	0.41	↓	–	–	–
10	Thymidine	1.63	1.26	↑	1.96	0.68	↓	1.66	0.75	↓
11	Succinic acid	1.80	1.40	↑	1.99	0.71	↓	1.81	0.67	↓
12	2-Ketobutyric acid	1.50	1.39	↑	1.50	0.73	↓	1.63	0.68	↓
13	Oxoglutaric acid	1.80	2.24	↑	1.82	0.54	↓	2.08	0.36	↓
14	Hydroxypyruvic acid	1.65	1.31	↑	1.55	0.81	↓	1.63	0.75	↓
15	L-Glutamic acid	1.77	1.56	↑	1.69	0.66	↓	2.02	0.55	↓
16	9,10-DHOME	2.00	3.05	↑	1.52	0.49	↓	–	–	–
17	Lupulone	1.71	5.06	↑	1.65	0.28	↓	–	–	–

↑The expression level was upregulated. ↓The expression level down-regulated; MS, mass spectrometry; m/z, mass-to-charge ratio; NEG, negative ion; POS, positive ion; RT, retention time; VIP, variable importance in projection.

### 3.4. Analysis of metabolic pathways after TAC rats intervention of Danhong injection

**Figure 8:**
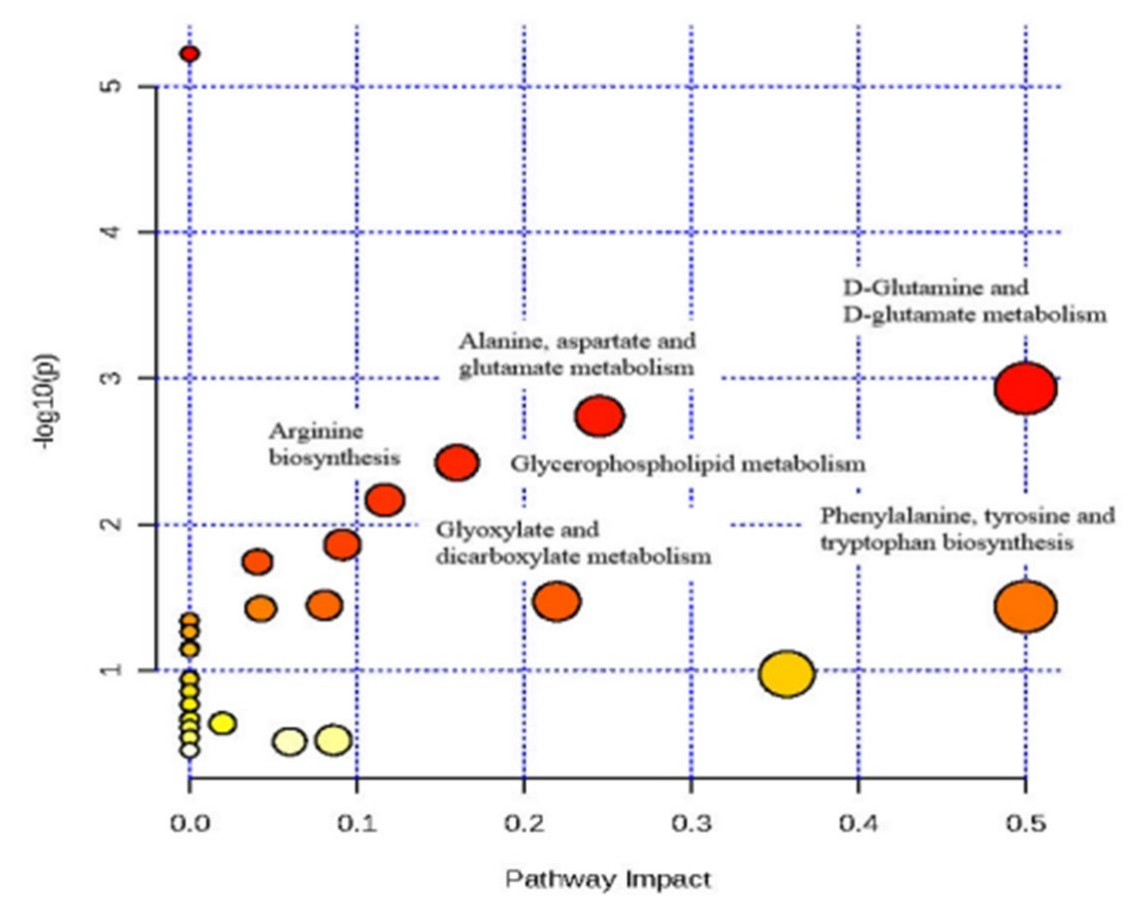
Analysis of metabolic pathways after TAC rats intervention of Danhong injection using MetPA.

An overview of the pathway analysis was shown in Figure 7. The 17 metabolites are involved in 27 different metabolic pathways, and 6 of the metabolic pathways were strongly relevant (raw *p* < 0.05 and pathway impact >0.1), including: (1) D-Glutamine and D-glutamate metabolism, (2) Phenylalanine, tyrosine and tryptophan genesis, (3) Alanine, aspartate and glutamate metabolism, (4) Glyoxylate and dicarboxylate metabolism, (5) Glycerophospholipid metabolism, and (6) Arginine biosynthesis (see Figure 8).

## 4. Discussion

Animal model is fundamental for HF research, the increased ventricular afterload caused by arteriosclerosis or hypertension can be better simulated by TAC, which makes TAC an ideal approach for replicating HF models. TAC increases left ventricular afterload by constricting the aortic arch diameter (26). The adaptive compensatory response of myocardial hypertrophy under long-term pressure overload is the key link in the progression to decompensation, which gradually leads to left ventricular enlargement and cardiac dysfunction, and ultimately progressed to heart failure (27). Two months after TAC, the inner diameter of the left ventricle of the model rats was significantly expanded, myocardial cells showed coagulation necrosis, and the biomarker of cardiac dysfunction NT-proBNP was significantly increased. However, in the case of abnormal ventricular dilation and decreased cardiac function, the TAC model did not show a significant decrease in cardiac output, but it did not adapt to the increased end-diastolic volume, whereas LVEF was decreased, these phenomena suggest that cardiac pumping function is significantly impaired. Consistent with previous publications, our data here demonstrated that DHI could protect against TAC-induced cardiac dysfunction, improving cardiac repair to maintain proper cardiac structure. Injury to the structure and function of the ventricle of the failing heart, the transfer of energy synthesis and utilization pathways, and various injury factors after heart failure are inevitably accompanied by changes in multi-level endogenous metabolic functions (28). Normaling and preserving certain metabolic functions may be key targets for HF treatment. Therefore, further application of UHPLC-QE-MS technique is helpful to clarify the metabolic influences of Danhong injection in the TAC model.

Simultaneously, our research utilized metabolomics analysis to provide a fresh perspective on understanding the mechanisms of DHI. While this approach to studying metabolic abnormalities in heart failure is relatively new, it has gained wide acceptance. Previous metabolomics studies have also identified metabolic disturbances, including disruptions in amino acid metabolism, lipid metabolism, and glucose metabolism in heart failure (29). These discoveries corroborate our results, further supporting the positive impact of DHI on metabolic irregularities. Among these six significant metabolic pathways identified in this study, most of them involved amino acid metabolism, and the pathway enrichment of L-glutamic acid was more significant. A variety of amino acids, including L-glutamic acid, are important components of proteins. L-glutamic acid and oxoglutaric acid played a role in controlling the metabolism of D-glutamine and D-glutamate, L-glutamic acid, succinic acid, and oxoglutaric acid were involved in alanine, aspartate, and glutamate metabolism, L-glutamic acid and oxoglutaric acid were involved in arginine biosynthesis, L-glutamic acid and hydroxypyruvic acid were involved in glyoxylate and dicarboxylate metabolism. Among the above metabolites and pathways, L-glutamic acid, glutamine, alanine, and arginine are amino acids found in proteins and encoded in the standard genetic code, which are critical players in promoting protein biosynthesis and stabilizing protein structure (30). Amino acid metabolites and associated pathways are disrupted, which results in the transfer of energy substrates in failing hearts, and subsequently enhanced amino acid metabolism to maintain energy supply to the heart (19). The disorder of amino acid metabolism will directly lead to the disorder of protein synthesis. This change is similar to the previous studies on salt-sensitive hypertensive heart failure. It reflects the common metabolic characteristics under left ventricular pressure overload. The emergence of the DHI intervention can correct the metabolic disorder of enhanced amino acid metabolism, help to promote protein synthesis and improve heart energy supply.

Lipid metabolism disorder is the major pathological mechanism of HF (31). Excessive lipid accumulation in blood will be deposited on the blood vessel wall, affecting blood flow and damaging blood vessels (32). There are 3 metabolites in the Glycerophospholipid metabolism. Glycerophosphocholine is one of the main forms of choline storage in the cytoplasm, which is formed by PC decomposition. PC is also known as lecithin, which is the active substance of cytomembrane and the source of lipid messengers such as LysoPC. PC is involved in lipid transport in organisms. LysoPC is converted from lecithin in the blood by lecithin cholesterol acyltransferase, LysoPC is low in most tissues, and up-regulation of LysoPC would lead to lysopc lysis (33). Oxygen-free radical-mediated peroxidation of lipids is considered to be a vital contributor to cell membrane destruction and damage. Particularly, it could modify lipids, mitochondrial DNA, and proteins, leading to mitochondrial membrane oxidative injury and mitochondrial structure disruption (34). Danhong injection can effectively improve phospholipid disorder, and its regulating effect on PC is better than that of trimetazidine, which is beneficial to the stability of biofilm and relieves vascular injury caused by lipid accumulation.

Notably, both DHI and TMZ showed significant improvements in cardiac function compared to the TAC model group. Specifically, left ventricular ejection fraction (LVEF) and left ventricular fractional shortening (LVFS) were significantly increased, while left ventricular end-diastolic volume (LVEDV) and NT-proBNP levels were notably decreased in both treatment groups compared to the TAC-induced model group. But the decreased HM/BM and LVM/BM induced by DHI treatment were more significant, suggesting that DHI may have a stronger effect on cardiac structure and function recovery than TMZ. These findings underscore the potential superiority of DHI as a treatment modality, providing robust support for its application in heart failure therapy.

There are some commonalities between Danhong injection and trimetazidine in regulating amino acid and phospholipid metabolism disorders in the TAC model, and this key biological information may be the underlying mechanism of drug action. Phenylalanine, which is catalyzed by phenylalanine hydroxylase, is the substrate for the phenylpropanoid biosynthesis pathway. Notably, together with tyrosine, phenylalanine participates in the production of a variety of neurotransmitters and hormones, which contribute to the metabolism of carbohydrates and lipids (35). Abnormal phospholipids can cause dysfunction of phenylalanine metabolism, which is a key risk factor for cardiovascular diseases. Research has shown that its expression level decreased gradually with the progression of myocardial ischemia (36). As shown in the current study, the phenylalanine levels in the TAC model were up-regulated, in contrast to the previous publication, which suggests that heart failure and coronary heart disease have inconsistent phenylalanine metabolic phenotypes in the ischemic state. Using phenylalanine and its related pathways as regulatory targets, it can exert an anti-ischemic effect (37) and improve cardiac ischemia and vascular injury (38).

This study determined the metabolic profile of heart failure induced by TAC and identified deferential metabolites by UHPLC-QE/MS analysis. We discovered 17 differential metabolites and six metabolic pathways. Danhong injection and trimetazidine both can regulate the disorder of amino acid and phospholipid metabolism to promote protein synthesis and energy supply and stabilize the cell membrane. Besides being a structural lipid of the cell membrane, PC plays an important role in lipid metabolism, abnormal lipid metabolism can cause dysregulation of L-Phenylalanine. PC and L-Phenylalanine reflect the damage degree of cell membrane structure. However, the effect of DHI was more specific to the regulation PC(18:3(6Z,9Z,12Z)/24:0), L-Phenylalanine and its associated pathways. All the regulatory effects help alleviate cell membrane damage caused by lipid peroxidation and other factors, particularly for the structure of mitochondrial lipids. Which in turn may have delayed the onset of cell apoptosis.

## 5. Conclusion

In this research, non-targeted metabolomics techniques of UHPLC-QE-MS were used to provide a more comprehensive picture of changes in metabolic profiles of pressure overload-induced heart failure and DHI intervention. Two biomarkers, PC(18:3(6Z,9Z,12Z)/24:0) and L-Phenylalanine, were identified for the first time as strong explanations for the significant effect of DHI. We gained new insights to further elucidate the mechanism and intervention strategy of the chain of cardiovascular events of “increased ventricular load – ventricular hypertrophy – heart failure.” These findings have provided a better understanding of CHF and highlighted the cardioprotective effects and mechanisms of DHI. It is believed that this study could contribute to the effective application in clinical practice of DHI intervention in heart failure.

## Data availability statement

The original contributions presented in the study are included in the article/Supplementary material, further inquiries can be directed to the corresponding author.

## Ethics statement

The animal study was approved by the Institutional Animal Care and Use Committee of the Hunan University of Chinese Medicine. The study was conducted in accordance with the local legislation and institutional requirements.

## Author contributions

LL: Writing – original draft. SZ: Writing – review & editing. JY: Data curation, Writing – review & editing. SH: Data curation, Writing – review & editing. ZH: Project administration, Writing – review & editing.

## Footnotes

^1^ http://www.metaboanalyst.ca

## References

[1] Heidenreich PA Bozkurt B Aguilar D Allen LA Byun JJ Colvin MM. 2022 AHA/ACC/HFSA guideline for the Management of Heart Failure: a report of the American College of Cardiology/American Heart Association joint committee on clinical practice guidelines. Circulation. (2022) 145:e895–e1032. doi: 10.1161/cir.0000000000001063, PMID: 35363499

[2] Boorsma EM Ter Maaten JM Damman K Dinh W Gustafsson F Goldsmith S. Congestion in heart failure: a contemporary look at physiology, diagnosis and treatment. Nat Rev Cardiol. (2020) 17:641–55. doi: 10.1038/s41569-020-0379-7, PMID: 32415147

[3] Wu C Chen F Huang S Zhang Z Wan J Zhang W. Progress on the role of traditional Chinese medicine in therapeutic angiogenesis of heart failure. J Ethnopharmacol. (2023) 301:115770. doi: 10.1016/j.jep.2022.115770, PMID: 36191661

[4] Wu Y Wang M Xu J Wei J Yang H. Signature network-based survey of the effects of a traditional Chinese medicine on heart failure. J Ethnopharmacol. (2022) 283:114750. doi: 10.1016/j.jep.2021.114750, PMID: 34662664

[5] Liu L Xu Y Su Z Man X Jiang Y Zhao L. Danhong injection Price trend and its utilization by coronary heart disease patients: evidence from hospital records in China. Front Pharmacol. (2022) 13:857167. doi: 10.3389/fphar.2022.857167, PMID: 35600876 PMC9114504

[6] Yao JY Zhi M Cao WT Huang Y Li CJ. Successful treatment with danhong injection for hepatic veno-occlusive disease. Hepato-Gastroenterology. (2011) 58:992–5. PMID: 21830430

[7] Zeng M Zhou H He Y Du H Yin J Hou Y. Danhong injection enhances the therapeutic effect of mannitol on hemispheric ischemic stroke by ameliorating blood-brain barrier disruption. Biomed Pharmacother. (2021) 142:112048. doi: 10.1016/j.biopha.2021.112048, PMID: 34435588

[8] Han L Wang C Yao D Wang B Zhang Z Liu J. Clinical efficacy and safety of Danhong injection for the treatment of chronic heart failure: a protocol for systematic review. Medicine (Baltimore). (2020) 99:e19526. doi: 10.1097/md.0000000000019526, PMID: 32243368 PMC7220450

[9] Feng X Li Y Wang Y Li L Little PJ Xu SW. Danhong injection in cardiovascular and cerebrovascular diseases: pharmacological actions, molecular mechanisms, and therapeutic potential. Pharmacol Res. (2019) 139:62–75. doi: 10.1016/j.phrs.2018.11.006, PMID: 30408571

[10] Yu T Li Y Yan M Zhang Z Yuan X Li S. Mechanism of Danhong injection in the treatment of arrhythmia based on network pharmacology, molecular docking, and in vitro experiments. Biomed Res Int. (2022) 2022:4336870–14. doi: 10.1155/2022/4336870, PMID: 35915792 PMC9338864

[11] Li C Yang J Tong X Zhao C He Y Wan H. Precursor ion scan enhanced rapid identification of the chemical constituents of Danhong injection by liquid chromatography-tandem mass spectrometry: an integrated strategy. J Chromatogr A. (2019) 1602:378–85. doi: 10.1016/j.chroma.2019.04.023, PMID: 31043229

[12] Wang Z Wan H Tong X He Y Yang J Zhang L. An integrative strategy for discovery of functional compound combination from traditional Chinese medicine: Danhong injection as a model. Biomed Pharmacother. (2021) 138:111451. doi: 10.1016/j.biopha.2021.111451, PMID: 33714107

[13] Li SN Li P Liu WH Shang JJ Qiu SL Zhou MX. Danhong injection enhances angiogenesis after myocardial infarction by activating MiR-126/ERK/VEGF pathway. Biomed Pharmacother. (2019) 120:109538. doi: 10.1016/j.biopha.2019.109538, PMID: 31629250

[14] Mu F Duan J Bian H Yin Y Zhu Y Wei G. Cardioprotective effects and mechanism of Radix Salviae miltiorrhizae and lignum Dalbergiae odoriferae on rat myocardial ischemia/reperfusion injury. Mol Med Rep. (2017) 16:1759–70. doi: 10.3892/mmr.2017.6821, PMID: 28656200 PMC5562082

[15] Yi X Wang F Feng Y Zhu J Wu Y. Danhong injection attenuates doxorubicin-induced cardiotoxicity in rats via suppression of apoptosis: network pharmacology analysis and experimental validation. Front Pharmacol. (2022) 13:929302. doi: 10.3389/fphar.2022.929302, PMID: 36071840 PMC9441549

[16] Li Y Sun X Zhang X Zhou H Wang D Xia Y. Functional damage of endothelial progenitor cells is attenuated by 14-3-3-n through inhibition of mitochondrial injury and oxidative stress. Cell Biol Int. (2021) 45:839–48. doi: 10.1002/cbin.11529, PMID: 33325040

[17] Murashige D Jang C Neinast M Edwards JJ Cowan A Hyman MC. Comprehensive quantification of fuel use by the failing and nonfailing human heart. Science. (2020) 370:364–8. doi: 10.1126/science.abc8861, PMID: 33060364 PMC7871704

[18] Zhou J Chen X Chen W Zhong L Cui M. Comprehensive plasma metabolomic and lipidomic analyses reveal potential biomarkers for heart failure. Mol Cell Biochem. (2021) 476:3449–60. doi: 10.1007/s11010-021-04159-5, PMID: 33974232

[19] Heggermont WA Papageorgiou AP Heymans S van Bilsen M. Metabolic support for the heart: complementary therapy for heart failure? Eur J Heart Fail. (2016) 18:1420–9. doi: 10.1002/ejhf.678 27813339

[20] Deng H Ma LL Kong FJ Qiao Z. Distinct phenotypes induced by different degrees of transverse aortic constriction in C57BL/6N mice. Front Cardiovasc Med. (2021) 8:641272. doi: 10.3389/fcvm.2021.641272, PMID: 33969009 PMC8100039

[21] Zhong S X X Zhang Q Hu S M Y Huang S. Pathological process observation and non-targeted metabolomics analysis of heart failure rat model established by transverse aortic constriction. Chin J Exp Tradit Med Formulae. (2022) 28:117–24. doi: 10.13422/j.cnki.syfjx.20211775

[22] Li L Zhong SJ Hu SY Cheng B Qiu H Hu ZX. Changes of gut microbiome composition and metabolites associated with hypertensive heart failure rats. BMC Microbiol. (2021) 21:141. doi: 10.1186/s12866-021-02202-5, PMID: 33952214 PMC8097775

[23] Wu Y Quan C Yang Y Liang Z Jiang W Li X. Renalase improves pressure overload-induced heart failure in rats by regulating extracellular signal-regulated protein kinase 1/2 signaling. Hypertens Res. (2021) 44:481–8. doi: 10.1038/s41440-020-00599-6, PMID: 33420473

[24] Ding S Chen M Liao Y Chen Q Lin X Chen S. Serum metabolic profiles of Chinese women with Perimenopausal obesity explored by the untargeted metabolomics approach. Front Endocrinol (Lausanne). (2021) 12:637317. doi: 10.3389/fendo.2021.637317, PMID: 34630316 PMC8498571

[25] Zhao SY Liu ZL Shu YS Wang ML He D Song ZQ. Chemotaxonomic classification applied to the identification of two closely-related Citrus TCMs using UPLC-Q-TOF-MS-based metabolomics. Molecules. (2017) 22:1721. doi: 10.3390/molecules22101721, PMID: 29027971 PMC6151587

[26] Richards DA Aronovitz MJ Calamaras TD Tam K Martin GL Liu P. Distinct phenotypes induced by three degrees of transverse aortic constriction in mice. Sci Rep. (2019) 9:5844. doi: 10.1038/s41598-019-42209-7, PMID: 30971724 PMC6458135

[27] Nakamura M Sadoshima J. Mechanisms of physiological and pathological cardiac hypertrophy. Nat Rev Cardiol. (2018) 15:387–407. doi: 10.1038/s41569-018-0007-y 29674714

[28] Peterzan MA Lygate CA Neubauer S Rider OJ. Metabolic remodeling in hypertrophied and failing myocardium: a review. Am J Physiol Heart Circ Physiol. (2017) 313:H597–h616. doi: 10.1152/ajpheart.00731.2016, PMID: 28646030

[29] Yuan Y Fan S Shu L Huang W Xie L Bi C. Exploration the mechanism of doxorubicin-induced heart failure in rats by integration of proteomics and metabolomics data. Front Pharmacol. (2020) 11:600561. doi: 10.3389/fphar.2020.600561, PMID: 33362553 PMC7758990

[30] de Vasconcelos DAA Giesbertz P Murata GM de Souza DR Fiamoncini J Duque-Guimaraes D. Myotube protein content associates with intracellular L-glutamine levels. Cell Physiol Biochem. (2019) 53:200–14. doi: 10.33594/000000130, PMID: 31287628

[31] Zhao P Wang Y Zhang L Zhang J Liu N Wang H. Mechanism of long non-coding RNA metastasis-associated lung adenocarcinoma transcript 1 in lipid metabolism and inflammation in heart failure. Int J Mol Med. (2021) 47:5. doi: 10.3892/ijmm.2020.4838, PMID: 33448307 PMC7834958

[32] Cone SJ Fuquay AT Litofsky JM Dement TC Carolan CA Hudson NE. Inherent fibrin fiber tension propels mechanisms of network clearance during fibrinolysis. Acta Biomater. (2020) 107:164–77. doi: 10.1016/j.actbio.2020.02.025, PMID: 32105833 PMC7160043

[33] Zeng C Wen B Hou G Lei L Mei Z Jia X. Lipidomics profiling reveals the role of glycerophospholipid metabolism in psoriasis. Gigascience. (2017) 6:1–11. doi: 10.1093/gigascience/gix087, PMID: 29046044 PMC5647792

[34] O'Farrell NJ Phelan JJ Feighery R Doyle B Picardo SL Ravi N. Differential expression profiles of oxidative stress levels, 8-oxo-dG and 4-HNE, in Barrett's esophagus compared to esophageal adenocarcinoma. Int J Mol Sci. (2019) 20:4449. doi: 10.3390/ijms20184449, PMID: 31509954 PMC6770156

[35] Pokorný V Štejfa V Havlín J Růžička K Fulem M. Heat capacities of l-histidine, l-phenylalanine, l-proline, l-tryptophan and l-tyrosine. Molecules. (2021) 26:4298. doi: 10.3390/molecules26144298, PMID: 34299573 PMC8305567

[36] Wang C Song C Zhang R Zhang D Yin D Zhu C. Metabolic phenotyping in subjects without coronary lesion, intermediate coronary lesions and serious coronary stenosis with acute myocardial infarction. Chin Circul J. (2020) 35:1192–5.

[37] Wang C Liu C Wang M Ma Q Li Y Wang T. UPLC-HRMS-based plasma Metabolomic profiling of novel biomarkers by treatment with KDZI in cerebral ischemia reperfusion rats. Molecules. (2018) 23:1315. doi: 10.3390/molecules23061315, PMID: 29849010 PMC6099697

[38] Heikal L Starr A Hussein D Prieto-Lloret J Aaronson P Dailey LA. L-phenylalanine restores vascular function in spontaneously hypertensive rats through activation of the GCH1-GFRP complex. JACC Basic Transl Sci. (2018) 3:366–77. doi: 10.1016/j.jacbts.2018.01.015, PMID: 29963647 PMC6018612

