# Supplementary figures and images for: Effect of Danhong injection on heart failure in rats evaluated by metabolomics
Source: Front Med (Lausanne). 2023 Oct 4;10:1259182. doi: 10.3389/fmed.2023.1259182 (PMC10582331; doi:10.3389/fmed.2023.1259182)

A

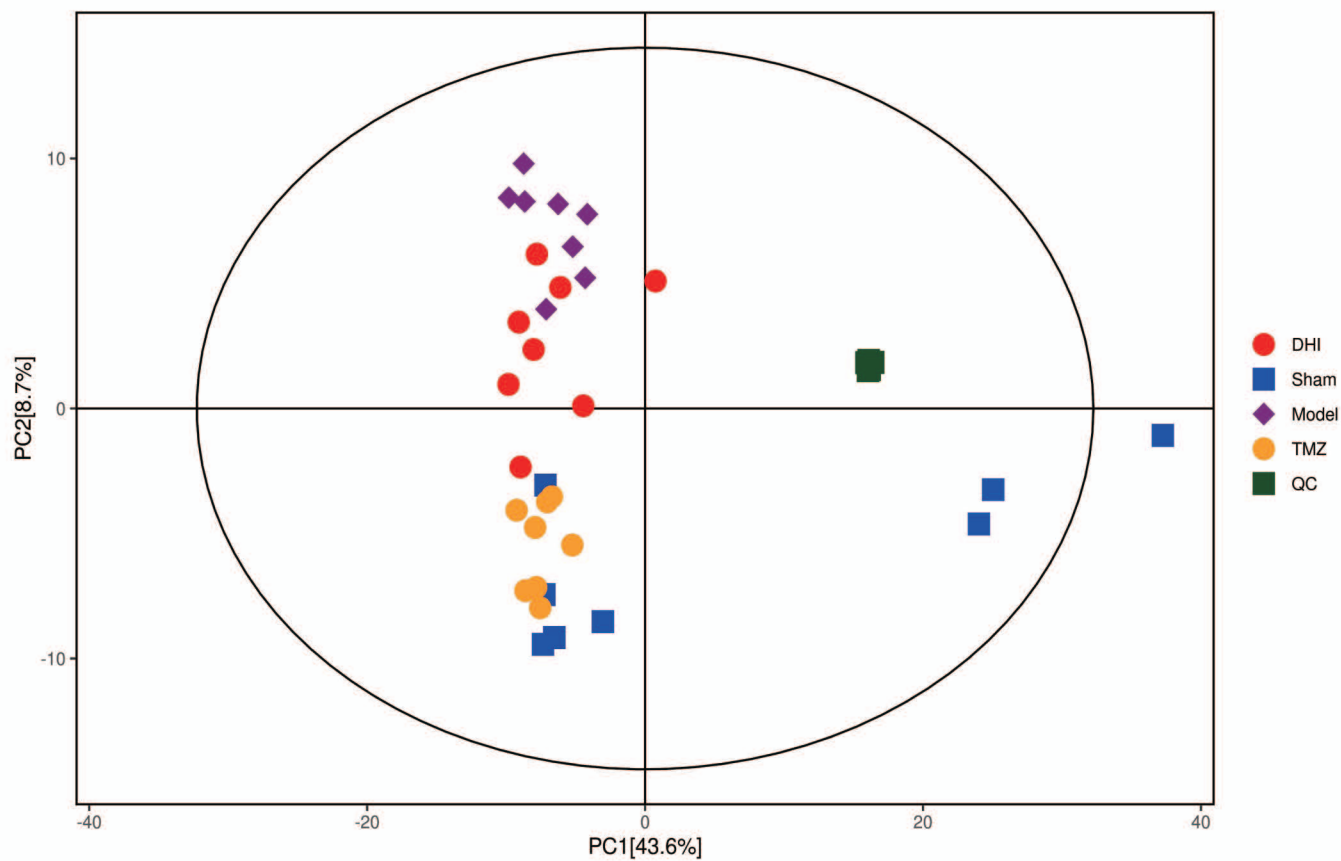

Supplement: Supplementary file 2 [file Image_1.PDF]

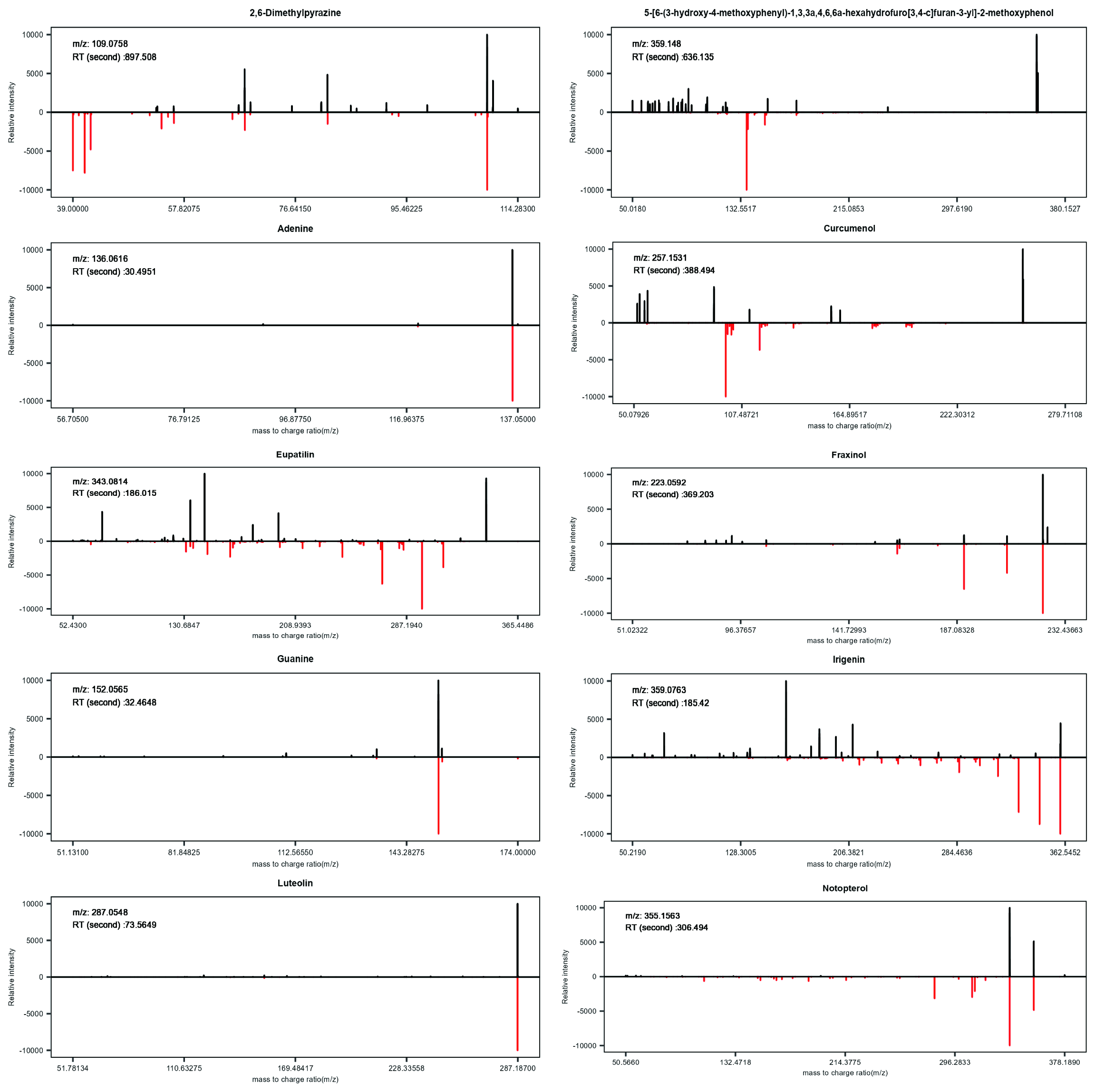

Supplement: Supplementary file 3 [file Image_2.TIF]
